# Patient Characteristics Associated With Chemotherapy-Induced Peripheral Neuropathy Severity in a Phase II Clinical Trial: A Retrospective Analysis

**DOI:** 10.1093/oncolo/oyad062

**Published:** 2023-03-27

**Authors:** Wanqing Iris Zhi, Nechama Dreyfus, Alexie Lessing, Marylou Galantino, Lauren Piulson, Kevin Liu Kot, Susan Li, Ting Bao

**Affiliations:** Breast Medicine Service, Solid Tumor Division, Department of Medicine, Memorial Sloan Kettering Cancer Center, New York, NY, USA; Weill Department of Medicine Cornell Medicine, New York, NY, USA; Department of Medicine, Renaissance School of Medicine, Stony Brook University, Stony Brook, NY, USA; Department of Medicine, School of Health Sciences, Stockton University, Galloway, NJ, USA; Integrative Medicine Service, Department of Medicine, Memorial Sloan Kettering Cancer Center, New York, NY, USA; Department of Medicine, Albert Einstein College of Medicine, Bronx, New York, NY, USA; Integrative Medicine Service, Department of Medicine, Memorial Sloan Kettering Cancer Center, New York, NY, USA; Breast Medicine Service, Solid Tumor Division, Department of Medicine, Memorial Sloan Kettering Cancer Center, New York, NY, USA; Integrative Medicine Service, Department of Medicine, Memorial Sloan Kettering Cancer Center, New York, NY, USA

**Keywords:** breast cancer, paclitaxel, peripheral neuropathy, body mass index

## Abstract

**Introduction:**

Chemotherapy-induced peripheral neuropathy (CIPN) can lead to chemotherapy dose reduction, delay, and discontinuation, and has limited effective prevention strategies. Our study aimed to identify patient characteristics associated with CIPN severity during weekly paclitaxel chemotherapy in people with early-stage breast cancer.

**Methods:**

We retrospectively collected baseline data including participants’ age, gender, race, body mass index (BMI), hemoglobin (regular and A1C), thyroid stimulating hormone, Vitamins (B6, B12, and D), anxiety, and depression up to 4 months prior to their first paclitaxel treatment. We also collected CIPN severity by Common Terminology Criteria for Adverse Events (CTCAE) after chemotherapy, chemotherapy relative dose density (RDI), disease recurrence, and mortality rate at the time of the analysis. Logistic regression was used for statistical analysis.

**Results:**

We extracted 105 participants’ baseline characteristics from electronic medical records. Baseline BMI was associated with CIPN severity (Odds Ratio [OR] 1.08; 95% CI, 1.01-1.16, *P* = .024). No significant correlations were observed in other covariates. At median follow-up (61 months), there were 12 (9.5%) breast cancer recurrences and six (5.7%) breast cancer-related deaths. Higher chemotherapy RDI was associated with improved disease-free survival (DFS, OR 1.025; 95% CI, 1.00-1.05; *P* = .028).

**Conclusions and Relevance:**

Baseline BMI may be a risk factor for CIPN and suboptimal chemotherapy delivery due to CIPN may negatively impact disease-free survival in patients with breast cancer. Further study is warranted to identify mitigating lifestyle factors to reduce incidences of CIPN during breast cancer treatment.

## Teaser

Chemotherapy-induced peripheral neuropathy (CIPN) is a common side effect of chemotherapy. This study aimed to identify patient characteristics associated with CIPN severity during weekly paclitaxel chemotherapy in people with early-stage breast cancer.

**Implications for Practice**

This retrospective analysis (*N* = 105) was conducted to identify patient characteristics associated with chemotherapy-induced peripheral neuropathy (CIPN) severity during weekly paclitaxel chemotherapy in people with early-stage breast cancer. Results showed that baseline body mass index may be a risk factor for CIPN and that suboptimal chemotherapy delivery due to CIPN may negatively impact disease-free survival in patients with breast cancer. This association between weight and CIPN offers clinicians a targetable characteristic to optimize and lessen the often life-long and treatment-limiting effects of CIPN.

## Introduction

Chemotherapy-induced peripheral neuropathy (CIPN) is a common and potentially debilitating side effect of chemotherapy agents such as platinum agents, taxanes, vinca alkaloids, and bortezomib.^1^ CIPN usually develops weeks to months after chemotherapy initiation with severity proportional to overall chemotherapy dose.^2^ Estimates of incidence range from 19% to more than 85%, and the highest reported incidence is with platinum-based agents, ranging from 70%-100%.^3,4^ Prevalence with platinum-based therapies ranges from 13% to 62% with a dose-dependent increase.^5^ Typically, taxane-­induced neuropathy (TIPN) causes a length-dependent neuropathy that can progress proximally. Common CIPN symptoms include numbness, tingling, altered touch, and paresthesia often triggered by cool or warmth.^2^ Although CIPN is predominantly a sensory neuropathy, motor, and autonomic changes can occur in severe CIPN, including shooting and/or burning pains, muscle weakness, orthostatic hypotension, constipation, and altered sexual or urinary function.^2^ CIPN symptoms can be long-lasting, persisting after chemotherapy completion and leading to increased psychological distress and fall risks.^6,7^ Studies have found that up to half of women with CIPN continued to report symptoms at six years post-treatment with resultant compromise in fall risk, function, and quality of life.^6-8^

Despite the high incidence and debilitating symptoms of CIPN, the American Society of Clinical Oncology (ASCO) 2020 CIPN guideline recommends that, during chemotherapy, the main intervention is chemotherapy dose reduction or discontinuation due to a lack of evidence of any effective CIPN prevention approaches.^9^ However, the consequence of chemotherapy dose reduction and discontinuation is suboptimal total drug delivery, which may worsen cancer-specific outcomes. In addition, currently, for patients with chronic painful CIPN after chemotherapy completion, duloxetine is the only agent that has been recommended for modest pain reduction.^9,10^

Additional treatment to reduce CIPN symptoms is needed and further emphasize the importance of CIPN prevention. Our group conducted a phase IIA trial that showed weekly acupuncture may be effective in preventing the progression of CIPN severity. In this retrospective study of this trial, we analyzed baseline characteristics associated with CIPN onset and progression during weekly paclitaxel in women with ­early-stage breast cancer to identify potentially modifiable risk factors that increase the risk of CIPN during chemotherapy, and to examine the effects of chemotherapy dose reduction on breast cancer-specific outcome.^11^

## Materials and Methods

### Participants

We consented to participants from May 2015 to March 2017 as part of screening in a phase IIA trial of weekly acupuncture intervention in women with early-stage breast cancer.^11^ Eligible participants were age 21 or older, diagnosed with stages I–-III breast cancer, and planned to receive 12 weekly sessions of neoadjuvant or adjuvant paclitaxel dosed 80 mg/m^2^, with an Eastern Cooperative Oncology Group (ECOG) performance status of 0-2 at recruitment. Concomitant trastuzumab and carboplatin treatments were allowed in this study.

We excluded participants with known metastatic disease; pre-existing peripheral neuropathy within 28 days of screening consent; or current use of anti-neuropathic pain agents including gabapentin, pregabalin, duloxetine, or glutamine. Informed consent was obtained from all participants prior to enrollment. The study was approved by the Memorial Sloan Kettering Cancer Center Institutional Review Board (ClinicalTrial.gov identifier NCT02364726) and we previously reported the primary outcome.^11^

### Data Collection

Baseline data including age, body mass index (BMI), gender, race, CIPN severity, hemoglobin, hemoglobin A1c, thyroid stimulating hormone (TSH), Vitamin B6, B12, and D levels, as well as the presence of anxiety or depression, were retrospectively extracted from the electronic medical record (EMR) on the first day of treatment or up to 4 months prior to chemotherapy initiation.

Severity of CIPN was defined according to the NCI-CTCAE v4.0 guidelines: grade 1 CIPN or paresthesia; grade 2 CIPN or moderate symptoms limiting instrumental activities of daily living; grade 3 CIPN or severe symptoms limiting self-care activities of daily living; and grade 4 CIPN, or life-threatening consequences for which urgent intervention is indicated.^12^ The CIPN severity was assessed by a primary physician and treating nurse at each week of chemotherapy. In this study, we used CIPN severity at the completion of paclitaxel for analysis.

Chemotherapy dosing was also extracted from each participant at the conclusion of the twelve-week treatment period in the EMR. Chemotherapy cumulative relative dose intensity was calculated by dividing the total chemotherapy dose received by the total chemotherapy dose planned. Per protocol, chemotherapy dosage adjustments were at the discretion of the treating provider.

Breast cancer recurrence and breast cancer-free survival were determined at the average follow-up period of 61 months (± 6 months) and confirmed at the time of retrospective chart review.

### Statistical Analysis

We performed statistical analysis using STATA software (Windows version 15.0, StatCorpLP, College Station, TX). Descriptive statistics of the study participants’ demographic and clinical variables were summarized using numbers and percentages. Univariate logistic regression was used to explore factors potentially associated with CIPN severity and disease-free status. All analyses were two-sided with a *P* < .05 indicating significance.

## Results

A total of 105 participants, who were enrolled from May 2015 to March 2017 in the screening phase of the pilot study, were included in the retrospective analysis (Table 1). The participants were all female, median age of 52 years (range 28-81 years). Baseline hemoglobin was collected on all participants with a median of 10.6 g/dL (interquartile range [IQR], 9.9–11.5 g/dL, with a normal range of 11.2-15.7 g/dL). HbA1C was collected on 10.5% of participants, median of 5.6 (IQR 5.1-13.4, with a normal range 4.0%-6.0%). TSH was collected on 35% of participants, median 1.295 (IQR 0.7-2.0, with normal range 0.550-4.780 ulU/m). Three participants were hypothyroid (2.9%). Participant’s median BMI was 26 (IQR 22.8-31.7). Eight participants had a diagnosis of depression at baseline (8%) and 10 had anxiety at baseline (10%). Three of these participants had a coexisting diagnosis of both depression and anxiety (3%). Ninety-nine of 105 (94%) participants received a dose of dense Adriamycin and cyclophosphamide (ddAC) for 4 cycles before starting weekly paclitaxel. Among 105 participants, 27 (26%) developed grade 2 CIPN during weekly paclitaxel and subsequently received weekly acupuncture treatment until paclitaxel completion.

**Table 1.** Participant characteristics (*n* = 105).

Age (median, IQR)	52 (43, 61)
Race, *n* (%)	
White	54 (52%)
Black	22 (20%)
Asian	11 (11%)
Other	7 (6%)
Unknown	11 (11%)
Hispanic ethnicity	13 (13%)
BMI (median, IQR)	26 (22.8, 31.7)
Breast cancer stage, *n* (%)	
I	16 (15%)
II	63 (60%)
III	26 (25%)
Receptor status, *n* (%)	
ER/PR+ and HER2−	14 (13%)
HER2+	73 (70%)
Triple negative	18 (17%)
Other chemo/anticancer therapy co-administered	
None	19 (18%)
Carboplatin	12 (11%)
Trastuzumab and/or pertuzumab	74 (71%)
Hemoglobin A1c (median, IQR), *n* = 11	5.6 (5.1-13.4)
Baseline hemoglobin (median, IQR)	10.6 (9.9-11.5)
Baseline TSH (median, IQR)	1.3 (0.7-2.0)
Vitamin B6 level (median, IQR), *n* = 5	16.6 (5.9-23)
Vitamin B12 level (median, IQR), *n* = 15	562 (404-2000)
Vitamin D level (median, IQR), *n* = 57	23 (6-1325)
Baseline anxiety, *n* (%)	10 (10%)
Baseline depression, *n* (%)	8 (8%)
Prior chemotherapy (adriamycin and cyclophosphamide), *n* (%)	99 (94%)
Breast cancer free survival at end of follow-up period (61 months ± 6 months), *n* (%)	93 (89%)
Relative paclitaxel chemotherapy dose intensity percent (dose received/preplanned total dose), (%)	91.7%
0%-25%	5 (5%)
26%-50%	0
51%-75%	8 (8%)
76%-99%	11 (10%)
100%	81 (77%)
Weekly acupuncture treatment for CIPN, *n* (%)	27 (26%)
CIPN grade by CTCAE 4.0, *n* (%)	
0	13 (13%)
1	56 (53%)
2	35 (33%)
3	1 (1%)

Abbreviations: CIPN, chemotherapy-induced peripheral neuropathy; CTCAE, Common Terminology Criteria for Adverse Events IQR, interquartile range; TSH, thyroid stimulating hormone;.

Of the baseline measures, there was a statistically significant correlation between BMI and CIPN severity (odds ratio [OR] 1.08; 95% CI, 1.01-1.16; *P* = .024, Table 2), suggesting that for each unit increase in BMI, there is an increased 8.2% odds of CIPN severity.

**Table 2.** Univariant analysis of baseline characteristics associated with CIPN severity.

	OR	95% CI	*P*-value
Age	1.04	0.99-1.10	.106
Race: Non-White	1.60	0.71-3.56	.25
BMI	1.08	1.01-1.16	.024
Hemoglobin A1c	0.88	0.57-1.36	.572

Abbreviations: BMI, body mass index CI, confidence interval; CIPN, chemotherapy-induced peripheral neuropathy; OR, odds ratio.

There was an association between the cumulative chemotherapy dose and disease-free survival (OR 1.026; 95% CI, 1.00-1.05; *P* = .028, Table 2). The cumulative chemotherapy dose was not statistically different between the acupuncture intervention group and the non-acupuncture intervention group (OR 1.01, 95% CI, 0.99-1.05; *P* = .26). Age, race, and HbA1C were not significantly associated with the risk of CIPN (Table 2).

## Discussion

In this study, we retrospectively identified possible characteristics associated with CIPN development during weekly paclitaxel chemotherapy treatment. We found that increased BMI was associated with a higher incidence of CIPN and that no other baseline characteristics were associated with CIPN severity. We also found a trend that higher chemotherapy dose intensity is associated with improved breast cancer free survival.

Taxanes are cornerstones of chemotherapy regimens for all stages of breast cancer. However, taxane-related CIPN remains a highly prevalent side effect that can jeopardize the optimal delivery of chemotherapy. Currently there is no effective strategy to prevent CIPN development during chemotherapy.^9^ The risk factors for development and progression of CIPN during chemotherapy remain poorly understood. Our results are consistent with prior findings that taxane and platinum-related CIPN correlate with higher BMI, waist circumference, and increased body surface area (BSA) in various malignancies.^13-18^ Our work adds to the evidence in patients with breast cancer exclusively, pulling together a more ­disease-specific population.^15,19^

The pathophysiology of this connection is not fully understood, but it is likely multifactorial. There is a suspected underdiagnosed prevalence of diabetes or prediabetes in the overweight population which in and of itself can influence nerve conduction.^20^ Evidence about whether diabetes is a risk factor for CIPN is mixed, leaning against an association. Of the 13 studies addressing this question, 4 found an association and 9 did not.^21^ Our study had a very limited sample size of Hb A1c and diabetes; we were not able to detect any statistical significance in its relationship to CIPN. We only have HbA1c values on 10% (*n* = 11) of our cohort, although 57% (*n* = 60) were overweight according to BMI (>25) which would indicate HbA1c screening. However, current literature on potential associations between CIPN and HbA1c largely fails to find an association.^16,22-25^ Additionally, an association in normoglycemic overweight patients with peripheral neuropathy is noted, indicating additional contributions between weight distribution and nerve injury.^16^ Another theory is that the increased chronic inflammation known to be associated with obesity may worsen the neuroinflammation that is thought to drive CIPN via increasing proinflammatory cytokines and immune signaling pathways.^26-28^

Those of African American race has a higher risk of CIPN and poor breast cancer outcomes.^29^ In our cohort, 20% of patients were African American. Among them, 41% developed grade 2 CIPN compared with 22% of white patients who developed grade 2 CIPN. Similarly, we observed that 38% of patients with Hispanic ethnicity had grade 2 CIPN compared with 27% of non-Hispanic patients. The difference was not statistically significant, most likely due to the small sample size. However, it is noteworthy that race and education level might indicate different perceptions of pain, in addition to genetic risks and health disparity factors, in CIPN detection and diagnosis.

Baseline anemia was previously found to be correlated with CIPN severity, though in our cohort, there was no association between hemoglobin level and CIPN.^14^ Age as a risk factor has had mixed results, with some studies finding a strong correlation and others, specifically in the breast cancer population, finding no such effect,^14,15^ consistent with this study. Pre-existing peripheral neuropathy due to other etiologies were excluded from our cohort.

Another major finding of our study was the association between weekly paclitaxel chemotherapy dose intensity and disease recurrence, as higher chemotherapy RDI was associated with improved disease-free survival (OR 1.025; 95% CI, 1.00-1.05; *P* = .028). This is consistent with a retrospective review of 874 breast cancer patients that showed a negative impact on overall survival (OS) with chemotherapy dose reductions.^30^ In the metastatic setting, a recent study found a significant correlation between OS with RDI < 85% in patients with a hormonal positive breast cancer and < 75% in triple-negative breast cancer patients.^31^ In early-stage breast cancer, RDI was found to be significant regarding OS when looking at neoadjuvant chemotherapy regimens, with a difference in 5-year OS between 91.2% and 76.3% when RDI was 85% and < 85%, respectively (*P* = .015).^32^ These data are consistent with other literature supporting the association of RDI with increased progression free survival (PFS) and decreased disease recurrence.^33-35^

Some weaknesses of our study include a small cohort size, and that it included all women with early-stage breast cancer and overall mild to moderate CIPN. All baseline laboratory values were inconsistent due to the nature of a retrospective analysis, in particular, baseline thyroid function tests, vitamin levels, and hemoglobin A1c. This limits our conclusions regarding thyroid function and diabetes status as risk factors for CIPN status.

## Conclusion

Despite these limitations, our study further supports that decreasing the chemotherapy regimen is associated with increased odds of disease progression, an effect not explicitly studied in recent work. Additionally, this study highlights an increased BMI as a risk factor for worsened CIPN. Better understanding and validation of these risks provide opportunities to identify those patients at risk for CIPN and interventions to mitigate the modifiable risks associated with this condition. CIPN remains a highly relevant clinical challenge and further research in risk characterization and optimization is needed.

## Data Availability

The data underlying this article will be shared on reasonable request to the corresponding author.
